# Antizyme Inhibitors in Polyamine Metabolism and Beyond: Physiopathological Implications

**DOI:** 10.3390/medsci6040089

**Published:** 2018-10-09

**Authors:** Bruno Ramos-Molina, Ana Lambertos, Rafael Peñafiel

**Affiliations:** 1 Department of Biochemistry and Molecular Biology B and Immunology, Faculty of Medicine, University of Murcia, 30100 Murcia, Spain; bruno.ramos@ibima.eu (B.R.-M.); ana.lambertos@um.es (A.L.); 2 Laboratory of Cellular and Molecular Endocrinology, Institute of Biomedical Research in Malaga (IBIMA), Virgen de la Victoria University Hospital, 29010 Málaga, Spain; 3 CIBER Physiopathology of Obesity and Nutrition (CIBERobn), Institute of Health Carlos III (ISCIII), 28029 Madrid, Spain; 4 Biomedical Research Institute of Murcia (IMIB), 30120 Murcia, Spain

**Keywords:** polyamines, polyamine metabolism, antizyme, antizyme inhibitors, ornithine decarboxylase

## Abstract

The intracellular levels of polyamines, cationic molecules involved in a myriad of cellular functions ranging from cellular growth, differentiation and apoptosis, is precisely regulated by antizymes and antizyme inhibitors via the modulation of the polyamine biosynthetic and transport systems. Antizymes, which are mainly activated upon high polyamine levels, inhibit ornithine decarboxylase (ODC), the key enzyme of the polyamine biosynthetic route, and exert a negative control of polyamine intake. Antizyme inhibitors (AZINs), which are proteins highly homologous to ODC, selectively interact with antizymes, preventing their action on ODC and the polyamine transport system. In this review, we will update the recent advances on the structural, cellular and physiological functions of AZINs, with particular emphasis on the action of these proteins in the regulation of polyamine metabolism. In addition, we will describe emerging evidence that suggests that AZINs may also have polyamine-independent effects on cells. Finally, we will discuss how the dysregulation of AZIN activity has been implicated in certain human pathologies such as cancer, fibrosis or neurodegenerative diseases.

## 1. Introduction

In mammalian cells, the control of the polyamine homeostasis is critical for the maintenance of cellular functions, since these molecules participate and modulate cellular processes such as cell growth, proliferation, differentiation and apoptosis [1,2,3]. Indeed, dysregulation of the intracellular polyamine levels has been observed in pathological conditions, ranging from cancer to inflammation, including neurological disorders [4,5,6,7,8,9,10,11]. The intracellular polyamine pool can be regulated by different mechanisms, including biosynthesis, catabolism and transport [12]. Ornithine decarboxylase (ODC), the rate-limiting enzyme of the polyamine biosynthetic pathway, catalyses the conversion of ornithine into the diamine putrescine by decarboxylation. Once putrescine is generated, the rest of the polyamines, spermidine and spermine, are produced as a consequence of the addition of aminopropyl groups from decarboxylated S-adenosylmethionine by the action of spermidine synthase and spermine synthase, respectively.

**Figure 1:**
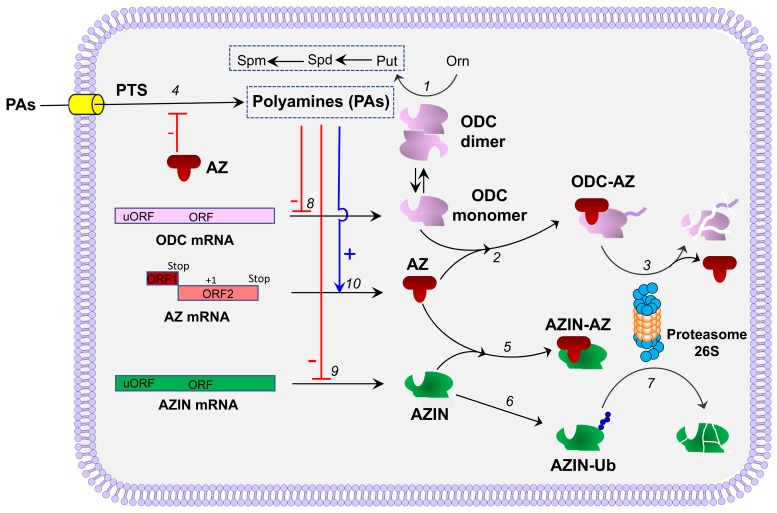
Antizymes and antizyme inhibitors in the control of polyamine homeostasis in mammalian cells. (**1**) The active ornithine decarboxylase (ODC) dimer catalyses the formation of putrescine (Put) from l-ornithine (Orn). Put serves as precursor in the synthesis of the major polyamines spermidine (Spd) and spermine (Spm). (**2**) Antizyme (AZ) inhibits ODC activity by binding to the ODC monomer. (**3**) The ODC-AZ complex interacts with the 26S proteasome where ODC is degraded and AZ is recycled. (**4**) AZ inhibits the polyamine transport system (PTS) at the plasma membrane by an unknown mechanism. (**5**) Antizyme inhibitor (AZIN) binds with higher affinity to AZ decreasing the negative effect of AZ on ODC and polyamine uptake. AZIN is ubiquitinated (**6**) and degraded (**7**) by the proteasome. The binding to AZ protects AZIN from its degradation by the proteasome. Polyamines (PAs) down-regulate the translation of ODC (**8**) and AZIN1 (**9**) mRNAs. (**10**) PAs stimulate the synthesis of AZs, by inducing the ribosomal frame-shifting at the stop codon of the ORF1 of AZ mRNA, allowing the translation of the ORF2.

**Figure 2:**
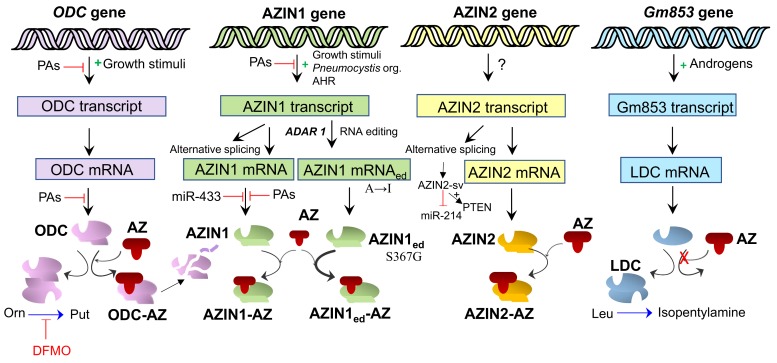
Scheme on the expression of ODC and its paralogues. The transcription of ODC and AZIN1 is up-regulated by different growth stimuli and down-regulated by polyamines (PAs). Translation of ODC mRNA is down-regulated by PAs and ODC activity and stability are decreased by AZ at the post-translational level. ODC activity is inhibited by α-difluoromethyl ornithine (DFMO). AZIN1 mRNA translation is inhibited by PAs and miR-433. PAs also affect AZIN1 splicing, and an edited form of AZIN1 with substitution of Ser367 by Gly has a higher affinity for AZ than AZIN1. AZIN2-sv is an lncRNA that interacts with miR-214 and activates phosphatase and tensin homolog (PTEN)*. Gm853* is a paralogous gene of ODC and AZINs, that does not interact with AZs and that catalyses the decarboxylation of l-leucine to produce isopentylamine (See Section 3.5). AHR: aryl hydrocarbon receptor; AZ: antizyme; LDC: leucine decarboxylase.

Ornithine decarboxylase activity is highly regulated in mammalian cells, being induced by different stimuli including oncogenes, hypoxic conditions or hormones [13]. Ornithine decarboxylase regulation is mediated by transcriptional, post-transcriptional, translational and post-translational mechanisms [3,13,14,15,16]. The post-translational control of ODC is mediated by a series of antagonistic proteins, antizymes (AZs) and antizyme inhibitors (AZINs) that down-regulate or up-regulate, respectively, the activity of ODC and the levels of polyamines [17] (Figure 1). In mammals, three different forms of ornithine decarboxylase antizymes (AZ1, AZ2 and AZ3) have been described [18]. Most studies on AZs (or OAZs) have been focused on AZ1, the first discovered antizyme. Although AZ2 and AZ3 share many functional properties with AZ1, they differ in their tissue and cellular distribution [19,20,21,22,23,24,25]. The synthesis of functional AZs is mediated by polyamines at the translational level. AZ is encoded by two partially overlapping open reading frames (ORFs), ORF1 and ORF2. At the end of the ORF1 there is a premature stop codon responsible for the synthesis of an incomplete form of AZ that cannot bind to ODC. Under high polyamine levels, a proportion of ribosomes that initiate at the start of ORF1 switch to the +1 reading frame at its last codon, skipping the stop codon, and proceed to decode the ORF2 and synthetize functional AZ [26,27] (Figure 1). The proportion of ribosomes that switch to the ORF2 frame depends on the intracellular polyamine concentration. Antizyme 1 inhibits ODC by interacting with the ODC monomer and therefore preventing the formation of active ODC homodimers, and induces the proteasomal degradation of ODC without ubiquitination [28,29]. Antizymes also affect the polyamine homeostasis by inhibiting the polyamine transport system at the plasma membrane [30,31,32], although the mechanism by which AZs inhibit the polyamine transporter is still unknown. Antizyme inhibitors (AZIN1 and AZIN2) are proteins homologous to ODC lacking enzymatic activity, that are able to interact with AZs even more efficiently than ODC, counteracting the negative effect of AZs on the biosynthesis of intracellular polyamines [17,33,34] (Figure 2). In addition, AZINs are able to positively modulate the uptake of extracellular polyamines, likely by preventing the inhibitory action of AZs on the polyamine transport system [35]. Interestingly, AZs and AZINs also regulate the uptake of other biogenic amines like agmatine, which could be also transported into the intracellular space by the canonical polyamine transporter [32]. Remarkably, the AZ/AZIN axis is not relevant for the regulation of the cellular uptake of cationic amino acids such as arginine, lysine or ornithine (Ramos-Molina et al., unpublished results), suggesting that both AZs and AZINs act specifically on the polyamine carrier and not on other organic cation transporters. In the subsequent sections, we will summarize the knowledge about these two proteins related with ODC regulation, as well as the most recent advances on our understanding of their pathophysiological implications.

## 2. Antizyme Inhibitor 1

The first antizyme inhibitor (here known as AZIN1) was originally characterized in rat liver extracts as a macromolecular inhibitor of the antizyme [36]. After its purification, it was demonstrated that it can bind to antizyme with higher affinity than ODC, releasing the enzyme from the ODC-antizyme complex [37,38]. The cloning of the rat and human genes contributed to deduce the protein sequence, showing that in spite of its high homology to ODC, AZIN1 is devoid of enzymatic activity [39,40]. This characteristic is shared by all AZIN1 orthologs studied, which have substitutions in several residues critical for ODC activity [41]. By negating the action of antizyme, AZIN1 can affect intracellular polyamine levels due to the concomitant increase of both ODC activity and polyamine uptake [42,43]. However, the possibility that AZIN1 could participate in the regulation of other processes by mechanisms unrelated to polyamines cannot be excluded.

### 2.1. Structural Aspects

Although initial studies suggested that AZIN1, like ODC, was able to form dimers, subsequent crystallographic and biochemical analyses revealed that under physiological conditions, AZIN1 exists as a monomer unable to bind pyridoxal 5-phosphate (a cofactor necessary for ODC activity), which could explain the lack of enzymatic activity and its high affinity to AZ [44]. More recently, it was described that the substitution of the residues Ser277, Ser331, Glu332 and Asp389 in AZIN1 for the corresponding residues of the putative dimer interface of ODC (Arg277, Tyr331, Asp332 and Tyr389, respectively) causes AZIN1 to behave as a dimer in solution [45]. Although both ODC and AZIN1 are proteins that can interact with AZ, AZIN1 has a higher AZ-binding affinity [42,46,47]. Mutational analyses demonstrated that the differences in certain residues in the AZ-binding element of ODC and AZIN1 are responsible for the differential AZ-binding affinities [48]. In fact, the substitution of residues N125 and M140 in ODC for lysines (corresponding residues in AZIN1) markedly increases the AZ-binding affinity to ODC. However, a more recent structural analysis of the AZIN1-AZ1 complex revealed that the residues A325 and S329, present in AZIN1 of all vertebrates, and that substitute N327 and Y331 in ODC may partially contribute to the higher affinity of AZIN1 for AZ1 [49]. Particularly interesting is the finding that the substitution of S367 by glycine leads to an AZIN1 variant with increased affinity for AZ1, likely by inducing a conformational change in its structure [50]. In addition, AZIN1 was able to interact not only with AZ1 but also with all members of the antizyme family, suggesting that AZIN1 may act as a general inhibitor of the function of antizymes [51]. On the other hand, AZIN1 variants unable to interact with AZs can still exert different cellular effects, suggesting that AZIN1 could also act by means of antizyme-independent mechanisms [52,53].

### 2.2. Tissue and Cellular Distribution

AZIN1, like ODC, is widely expressed as evidenced by the analysis of AZIN1 mRNA levels in different rat and mouse studies [39,54,55]. Although several types of AZIN1 mRNA have been found both in human and rodents, the ORF remains unaltered in most cases [39,40,56]. More recently, multiple forms of *Azin1* transcripts formed by alternative splicing and initiation of transcription from putative alternative start sites were reported in mice [57]. One of the novel splice variants encoded a truncated form of AZIN1 whose functional significance remains to be clarified. Remarkably, an edited transcript of AZIN1 was firstly detected in human hepatocellular carcinoma [50]. Although AZIN1 editing was also detected in healthy liver tissues, the level of editing increased with the pathological behaviour of the tumor. The AZIN1 mRNA A→I editing, which is mediated by a double stranded RNA specific adenosine deaminase (ADAR1), resulted in a Ser to Gly change at the residue 367 of AZIN1 protein that, as commented above, increased the affinity of AZIN1 for AZ1 [50]. At the cellular level AZIN1, like AZ1, has been found to be located in the centrosomes, where it can modulate centriole amplification. In fact, silencing of AZIN1 reduced centrosome abnormalities, whereas its overexpression produced centrosome overduplication [58]. In other cases, such as in HEK293T and COS7, cells transfected with AZIN1, AZIN1 protein were mainly located in the nuclei [59]. Changes in the subcellular location have been observed along the cell cycle or during development. Thus, AZIN1 was found to be present in the cytoplasm of hepatoma tissue culture (HTC) cells during interphase, and together with AZ1, at centrosomes during mitosis [60]. During the cell cycle AZIN1 was mainly accumulated at the early G1 period, likely to increase ODC activity, and in the G2/M phase, and its suppression increased the number of binucleated cells [60]. In addition, translocations between nucleus and cytoplasm were reported to take place during murine mammary gland development [61]. Together, these data suggest an important role of AZIN1 in cell division and differentiation.

### 2.3. Synthesis and Degradation

Early studies indicated that the amount of AZIN1 increased in rat liver in response to different nutritional stimuli [38]. More recent studies have shown that AZIN1 expression appears to be regulated at several levels by different factors related to cell growth. In mouse fibroblasts, *Azin1* mRNA content increased significantly following growth stimulation much earlier than the increase of ODC transcripts under the same conditions [56]. Furthermore, in breast cancer cells, AZIN1 was transiently up-regulated after induction of cell proliferation by diluting cells in fresh medium [62]. In alveolar macrophages, *Pneumocystis* organisms were found to induce AZIN1 expression, suggesting that this increase is related to the high polyamine content of these cells during pneumonia [63]. In fact, in mouse embryonic fibroblasts the inhibition of polyamine synthesis by α-difluoromethyl ornithine (DFMO), an irreversible ODC inhibitor, increased the *Azin1* mRNA levels, whereas the addition of polyamines resulted in opposite effects [57]. Remarkably, this effect of polyamines did not only affect *Azin1* transcription but also its splicing pattern. In addition, other studies have suggested that polyamines may mediate the repression of *Azin1* through its action at translational level on the short functional upstream ORF (uORF) existing in the *Azin1* mRNA [64]. Another element, a long-looped quadruplex detected in the 5’ untranslated region (5’UTR) in the *Azin1* mRNA, has been postulated as a regulator and sensor of polyamine levels [65]). Interestingly, it was reported that AZIN1 expression is also down-regulated by miR-433 [66], and therefore by sequestration of this miRNA, *Azin1* mRNA could indirectly mediate the expression of other genes targeted by miR-433. Recently it has been also reported that iron depletion up-regulates AZIN1 protein expression, although the mechanism is currently unknown [67].

Antizyme inhibitor 1, like its homologous ODC, is a short-lived protein, but in contrast to ODC, it is degraded by the proteasome by an ubiquitin-dependent mechanism [68]. Furthermore, although AZIN1 binds tightly to AZ1, this does not accelerate its degradation as in the case of ODC. Rather, antizyme binding stabilizes AZIN1 by preventing its ubiquitination [69].

### 2.4. Physiological Role

Antizyme inhibitor 1 is essential for survival, since transgenic mice with disruption of the gene died at birth showing abnormal liver morphology, slightly reduced body weight and decreased polyamine levels in several tissues [54]. This finding was in agreement with the notion, above commented, that AZIN1 exerts a positive effect on intracellular polyamine levels by repressing the inhibitory action of antizymes on the polyamine biosynthetic and transport pathways. Since there is ample evidence suggesting that increasing polyamine levels stimulates cell growth [2,3], it was postulated that AZIN1 could have a role in cell proliferation. This possibility was later confirmed by different experiments in both normal and transformed cells. Thus, stimulation of cell proliferation in both normal and cancer cells was associated to increased expression of AZIN1 [56,62,69]. In addition, induced overexpression of AZIN1 stimulates the growth, survival and oncogenic potential of tumor and non-tumor cells [42,52,62,69]. Conversely, silencing expression of AZIN1 by using RNA interference technology reduced intracellular polyamine levels and decreased the proliferation of cultured cancer cells [70] and prostate tumor growth in vivo [71]. Although most of the effects of AZIN1 appear to be related to its capacity to interfere the action of AZ1, it has been also reported that AZIN1 is able to interact directly with the cell cycle regulator cyclin D1, preventing the degradation of this cyclin [52]. It is then likely that AZIN1 may also affect cell proliferation by antizyme-independent mechanisms. In fact, the transcriptional profile of livers from *Azin1* knock-out mice at 19th day of gestation showed marked changes related to those of wild type mice, affecting genes related with cell cycle control and proliferation [72]. Finally, emerging evidence has suggested that AZIN1 may have also functions not directly related with cell proliferation. For instance, the similarity in the expression pattern between AZIN1 and certain reproductive related genes in the hypothalamus, ovary and uterus during the rat oestrous cycle [73] or in the avian ovarian follicles [74], suggested a possible role of AZIN1 in reproductive physiology. Transcriptomic studies have also revealed that *Azin1* is one of the genes that are consistently up-regulated by glucocorticoids in the brain [75]. Additionally, either the overexpression or the knock down of *Azin1*, in neurons of the paraventricular and supraoptic nuclei of the hypothalamus, revealed that AZIN1 could be important for the transcriptional regulation of arginine vasopressin [76].

### 2.5. Antizyme Inhibitor 1: Overexpression and RNA Editing in Cancer

Following the initial study showing that AZIN1 was highly expressed in human gastric tumor cells [77], current information available from the Oncomine (https://www.oncomine.org) or Gene Expression Profiling Interactive Analysis (GEPIA) (http://gepia.cancer-pku.cn) databases have revealed that AZIN1 is up-regulated in many different types of human malignancies. These findings are in agreement with experimental studies reporting that transformed NIH-3T3 fibroblast cells with over-expression of AZIN1 generated tumors after injection into nude mice [42], and that the overexpression of AZIN1 in rat prostate carcinoma cells enhanced their ability to grow in soft agar [52]. On the other hand, the knocking down of AZIN1 using shRNA in both human and rat prostate cancer cell lines decreased the ability of these cells to form tumors in vivo, after subcutaneous injection into nude mice [71].

Interestingly, a series of new findings have established that the RNA editing of AZIN1 may be a potential driver in the pathogenesis of human cancers. In the seminal paper [50], transcriptomic sequencing of several human hepatocellular carcinomas revealed that adenosine-to-inosine (A→I) RNA editing of AZIN1 was increased in tumors with respect to healthy liver tissue. This specific editing of the AZIN1 transcript resulted in the substitution of serine by glycine at residue 367 of human AZIN1. Remarkably, this change increased the affinity of AZIN1 toward antizyme, and induced the translocation of AZIN1 from the cytoplasm to the nucleus. When hepatocellular carcinoma cell lines were transduced with lentivirus carrying the edited version of AZIN1, they showed accelerated growth rates and higher frequency of colony formation. Edited AZIN1 cells also showed enhanced in vivo tumorigenic capacity [50]. This kind of epigenetic modification reported in liver tumors has also been described in other types of cancer. In oesophageal squamous cell carcinomas, overexpression of ADAR1 (the adenosine deaminase that converts adenosine into inosine acting on dsRNA) due to gene amplification was detected [78]. This resulted in hyper-editing of AZIN1, which conferred a gain-of-function phenotype associated with a more aggressive tumor behavior [78]. A close association between ADAR overexpression and AZIN1 editing was also observed in several non-small-cell lung cancer patient samples and lung cancer cell lines [79]. In these tumors, AZIN1 protein expression was higher in tumors with edited AZIN1 than in those with non-edited AZIN1. In lung cancer cell lines AZIN1 RNA editing induced proliferation, invasion and migration, both in vitro and in vivo [79]. More recently, AZIN1 RNA editing was analyzed in 392 colorectal tissues from multiple independent colorectal cancer patient cohorts. This study showed that AZIN1 RNA edited levels were higher in cancer tissue compared to normal mucosa, and that high levels of editing of AZIN1 may be considered as a prognostic factor for disease-free survival and overall survival, and also as an independent risk factor for lymph node and distant metastasis [80]. According to all these findings, it has been postulated that AZIN1 might be a critical target for cancer therapy [81]. However, since in these studies polyamine levels were not determined, it is difficult to ascertain whether all the changes observed could be explained by an increase in polyamine levels, or are rather linked to interactions with proteins not directly related with polyamine metabolism. It should be also necessary to know the influence of the hyper-editing of other genes in the changes observed in the cancer cells with overexpression of ADAR enzymes. The generation of conditional mouse models with wild type and edited *AZIN1* genes would provide valuable information on the relationship between AZIN1 and AZIN1 editing with tumor development. Interestingly, a recent report revealed that the aryl hydrocarbon receptor (AHR), acting as a transcriptional factor, activated the expression of both AZIN1 and ODC [82]. Moreover, in this work a new drug (clofazimine) was identified as a potent AHR antagonist that inhibited polyamine biosynthesis, decreased intracellular polyamine content and the growth of human multiple myeloma [82]. Consequently, targeting of AZIN1 through AHR inhibition appears as a promising strategy for cancer therapy.

### 2.6. Antizyme Inhibitor 1 and Fibrogenesis

Different studies have revealed the existence of a correlation between AZIN1 and fibrogenic processes in liver and kidney. Hepatic fibrosis is related with the conversion of hepatic stellate cells into myofibroblast-like cells. A single nucleotide polymorphism (SNP) in the AZIN1 gene seemed to be associated with slower rate of hepatic fibrosis in chronic viral hepatitis C [53]. This SNP increased the formation of a spliced variant of AZIN1 mRNA (AZIN1 SV2) that encoded a truncated version of the AZIN1 protein. In addition, the transfection of LX2 stellate hepatic cells with the variant AZIN1 SV2 inhibited fibrogenic gene expression through a polyamine-independent mechanism [53]. In another study, an association between a SNP of AZIN1 and liver cirrhosis risk in Chinese hepatitis B patients was also postulated [83].

Antizyme inhibitor 1 has also been implicated in the regulation of renal fibrosis, since AZIN1 overexpression suppressed transforming growth factor β (TGF-β)/Smad3 signalling pathway, a major player in tissue fibrosis [66]. In this process, micro RNA miR-433 was identified as an important component, since miR-433 overexpression suppressed *Azin1* expression and enhanced TGF-β1-induced fibrosis, whereas *Azin1* overexpression suppressed TGF-β signalling and the fibrotic response [66]. In this context, increased *Azin1* expression has been also correlated with the amelioration of renal fibrosis associated to diabetic nephropathy [84].

## 3. Antizyme Inhibitor 2

The second member of the AZIN family, named AZIN2, was identified in mouse and human [85,86]. This gene, earlier identified as *ODCp* or *ODC-like*, was mainly expressed in human brain and testes, as assessed by dot blot analysis [87]. Quantification of the expression levels of *Azin2* in mouse tissues by reverse transcription-polymerase chain reaction (RT-PCR) showed that its expression was about 23-fold higher than *Azin1* in the testes and 6-fold in the brain [88]. Due to this specific cellular distribution, it was initially suggested that AZIN2 could play a role in terminal differentiation rather than in cell proliferation.

### 3.1. Structural and Functional Aspects

Despite its homology with ODC, AZIN2 is not capable of decarboxylating ornithine [84,85,86], probably due to differences in some critical residues required for the enzymatic activity. On the other hand, AZIN2 binds efficiently to the three AZs, counteracting the negative regulation of these proteins on ODC and the polyamine uptake [35,85,86], although its binding to AZ1 and AZ3 appears to be less efficient than AZIN1 [88]. Regarding to this point, human AZIN2 has substitutions in the corresponding residues A325 and S329 of AZIN1 (N328 and F333 in AZIN2, respectively) that were shown to be relevant for the binding to AZs [49]. Sequence analogy with ODC and AZIN1 is high, mainly in the central part of the molecule and especially in the denominated antizyme-binding element (AZBE) [34]. Although the AZBE site is delimited between the residues 110 and 145, the specific residues involved in the direct interaction with AZs has not been fully identified. By multi-alignment sequence analysis of the AZBE region of AZIN2 orthologues and those corresponding paralogues provided by genome database, five conserved residues (K116, A124, E139, L140, and K142) were identified [89]. Whereas single mutations in these residues did not affect AZ binding, double or triple mutants markedly reduced the affinity of AZIN2 towards AZ1 [90]. AZIN2, in contrast to ODC, does not form homodimers, although its predicted monomeric tertiary structure was similar to that of ODC [90].

Antizyme inhibitor 2 is a short-lived protein. In human embryonic kidney (HEK) 293T-transfected cells the half-life of AZIN2 was much lower (≈90 min) than that ODC (>8 h) [90], but AZIN2 was less labile than AZIN1 [88,90]. The degradation of AZIN2 was reduced by the presence of any of the three AZs paralogues [90]. Interestingly, AZIN2 increased the stability of the three antizymes, as it was shown by co-transfections experiments [90]. Like AZIN1, it is degraded in an ubiquitin-dependent manner by a process that is inhibited by AZ1 [86,88], whereas in NIH-3T3 cells stably overexpressing *Azin2* the degradation of AZIN2 was inhibited by the proteasome inhibitor MG132 [88], and in transiently transfected HEK293T cells the effect of MG132 on AZIN2 was not so evident as in the cases of ODC and AZIN1 under the same experimental conditions [90]. In addition, the protective effect produced by inhibitors of the lysosomal degradative pathway suggested that AZIN2 may be also degraded by an alternative route to that of proteasome [90].

Regarding the subcellular localization of AZIN2 in both HEK293T and COS7 cells overexpressing AZIN2, the protein was mainly present in the ER-Golgi intermediate compartment (ERGIC) and in the *cis*-Golgi network [59]. In human neural Paju cells, immunostaining with rabbit antisera against AZIN2 revealed a vesicle-like expression pattern in the cytoplasm [91]. Co-localization studies with other subcellular markers in Paju cells transfected with AZIN2-FLAG indicated that AZIN2 localizes in the post-Golgi vesicular compartments of the secretory pathway [91]. Interestingly, co-expression of AZIN2 with any member of the AZs induced a shift of AZIN2 from the ERGIC to the cytosol [59]. Furthermore, whereas the deletion of the AZBE region did not alter AZIN2 location, the ablation of its N-terminal region abrogated the incorporation of the mutated AZIN2 to the ERGIC complex, revealing that this part of the protein plays a relevant role for the vesicular localization of AZIN2 [59]. Importantly, RNAi-mediated knockdown of AZIN2 produced a distorted morphology of the trans-Golgi network, although the functional impact of this change was not addressed [91]. Furthermore, AZIN2 has been detected by immunohistochemistry in granular or vesicle-like structures of the cytoplasm in different cell types, including neurons, human neural-crest-derived tumour cells, mast cells, ovarian hilus, corpus luteum and Leydig cells [92,93,94]. In mast cells, AZIN2 is specifically accumulated in serotonin-containing granules where its expression is rapidly induced after activation with phorbol 12-myristate 3-acetate or calcium ionophore A23187 [92]. This activation was associated with changes in the intracellular distribution of AZIN2, which relocated from the cytoplasm and nucleus to the peripheral areas of the cells, suggesting that AZIN2 might play a role in exocitosis [92].

Although transfection experiments clearly showed that AZIN2 stimulates ODC activity and polyamine uptake [35,85,86,88] little is known about its effects on polyamine levels in vivo. However, NIH-3T3 cells stably overexpressing AZIN2 grow more rapidly than control cells, but less than cells overexpressing AZIN1, indicating that AZIN2 provides cells some growth advantage [88]. Nowadays, the analysis of expression of AZIN2 in cancer database reveals that this gene is not up-regulated in most of the types of cancer examined (GEPIA (http://gepia.cancer-pku.cn). All these findings, together with the fact that AZIN2 is widely expressed in differentiated cells, support the contention that its major physiological role, in contrast to AZIN1, does not appear to be related with the stimulation of cell proliferation. In fact, transgenic mice with deleted *Azin2* gene are viable [95], in clear difference with *Azin1* knockout mutants [54].

### 3.2. Antizyme Inhibitor 2 in the Central Nervous System

Polyamines and their metabolic enzymes are present in the mammalian brain, showing a specific regional distribution [96,97], including the presence of antizyme in association with ODC [98,99]. Current evidence clearly indicates that AZIN2 is present in the brain showing a complex regional distribution, and that the expression pattern may be altered in some neurological diseases. Initial experiments detected high amounts of AZIN2/ODCp mRNA in different parts of the human adult brain, including cerebral cortex, cerebellum, hippocampus, substantia nigra, thalamus, corpus callosum and spinal cord [86]. A subsequent semiquantitative RT-PCR analysis using primers for AZIN2 mRNA detected the expression of AZIN2 in different regions of the rat brain (frontal cortex, hippocampus, hypothalamus, locus coeruleus, medulla and striatum) and the human brain (frontal cortex, hippocampus and nucleus accumbens), and in cultured rat neuronal cells (neurons, PC12, astrocytes and glioma cells) [100]. More recently, by using in situ hybridization and immunohistochemistry, a robust expression of AZIN2 was found in the soma and axon of human neurons from different areas of the central nervous system [94]. In this study, the subcellular localization of AZIN2 was dependent on the type of neuronal cell examined. In pyramidal neurons of the frontal cortex, AZIN2 staining was located in granular or vesicle-like structures, whereas in the Purkinje cells of the cerebellum the staining pattern was more diffuse. In the pyramidal neurons of the cortex, AZIN2 co-localized with the *N*-methyl-d-aspartate (NMDA) glutamate receptor. Interestingly, in some neurons of brains affected of Alzheimer disease, a robust expression of AZIN2 was observed [94]. We recently analysed the expression of AZIN2 in the brain of transgenic mice carrying an *Azin2-lacZ* construct under the control of the *Azin2* endogenous promoter. X-Gal staining of brain sections revealed a strong but heterogeneous AZIN2 expression pattern [101]. Labeled neurons showed various sized vesicles full of the LacZ reaction product, and some axons tracts also showed β-galactosidase staining in varying degrees. AZIN2 expression predominantly coincided with cholinergic and glutamatergic cells, and occasionally corresponded to GABAergic and glycinergic cells [101]. In spite of all these findings, the plausible physiological role of AZIN2 in the central nervous system remains to be elucidated.

A controversial issue on AZIN2/ODCp in the brain was related with the assertion that this gene encoded for an arginine decarboxylase (ADC) [102], the enzyme that in plants and bacteria catalyses the formation of agmatine from l-arginine. Although other studies from several independent laboratories clearly demonstrated that the product of this gene was devoid of ADC activity [85,86,103] the term ADC is still present in mammalian gene databanks, as a gene synonym of AZIN2. Even more, some of the effects ascribed to ADC in some expression or transfection experiments should be credited in all likelihood to AZIN2 [104,105,106,107,108,109,110].

### 3.3. Antizyme Inhibitor 2 in Reproductive Tissues

The specific role of polyamines in reproductive tissues is not fully understood [111]. As commented above, preliminary studies showed a high expression of AZIN2 in the testes of adult humans and mice [85,87]. Subsequent comparative analyses revealed that the testis is the tissue with the highest levels of *Azin2* mRNA among the murine tissues examined [55]. Importantly, *Azin2* mRNA was undetectable in the testes of newborn mice, but it markedly increased along the first wave of spermatogenesis to reach constant values after the 7th week of age [112]. Interestingly, in situ RNA hybridization and immunochemical analyses revealed that mouse *Azin2* in mainly expressed in the inner part of the seminiferous tubules, where spermatids at different stages of differentiation and spermatozoa are located [112]. The fact that this spatial and temporal expression pattern was similar to that of *Az3*, the testis-specific antizyme isoform [21,22], suggested that AZIN2 may have a role in spermiogenesis, likely by affecting polyamine homeostasis [112]. However, unlike *Az3* knockout mice, which were infertile despite showing unaffected testicular polyamine [113], *Azin2* knockout mice were fertile [94]. It is plausible that the functions of these two proteins, apparently antagonists, could be related with targeting other proteins not related to polyamine metabolism. In fact, AZ3 can interact with gametogenetin, a testicular protein [114], and with MYPT3, a regulatory protein of the protein phosphatases PP1β and PP1γ2 in the testis [115].

On the other hand, immunochemical analyses revealed the presence of AZIN2 protein in the steroidogenic cells of human gonads [93]. Thus, a robust expression of AZIN2 was found in the testicular Leydig cells, and in ovarian luteinized cells, suggesting a role of AZIN2 in steroidogenesis [93]. In this context, *Azin2* knockout mice showed decreased testosterone levels in plasma and testis, as well as decreased sperm motility [116]. Furthermore, in addition to mammalian gonads, AZIN2 expression has been also reported in ovarian follicles in the goose [117]. All these results support that AZIN2 may play a relevant role in the reproductive system.

### 3.4. Antizyme Inhibitor 2: Expression in Other Tissues

Although the initial studies on AZIN2 expression in mammalian organs appeared to indicate an exclusive distribution of the AZIN2 mRNA in brain and testis, in more recent analyses by real-time RT-PCR lower levels of AZIN2 messenger were detected in other mouse tissues, including epididymis, pancreas, adrenal gland, kidney, lung, heart, intestine and liver [55]. Interestingly, the comparison of the relative expression of *Azin2* with *Azin1* in each tissue revealed that *Azin2* mRNA was more abundant than *Azin1* mRNA in testis, adrenal gland, lung, brain and epididymis. In addition, the analysis of the gene expression in different renal zones revealed that *Azin2* mRNA levels were lower than those of *Azin1* in all zones, without differences between male and female kidneys [118]. More recently, a novel lncRNA that is up-regulated in human adult hearts was identified as a splice variant of the AZIN2 gene [119]. It has been postulated that this AZIN2-sv transcript decreases the activation of the PI3K/Akt signalling pathway by directly activating phosphatase and tensin homolog (PTEN), and also by negating the negative effect of the miR-214 on PTEN expression. Interestingly, knockdown of AZIN2-sv attenuated ventricular remodelling and improved cardiac function after myocardial infarction of adult rats [119]. Whether AZIN2 mRNA, which contains as AZIN2-sv the complementary sequence to the seed sequence of miR-214, is able to mimic some of these effects, remains to be tested.

By immunological characterization, the AZIN2 protein has been detected in the mast cells of sections of human skin samples from patients with cutaneous mastocytosis and in the cytoplasm and nuclei of different human and murine mast cell lines [92]. Remarkably, AZIN2 was selectively expressed in serotonin-containing mast cell granules, in which serotonin release was polyamine dependent [92]. Additional information has been provided by studies using bone marrow-derived mast cells from both wild type and transgenic *Azin2* knockout mice. Compared to wild-type controls, mast cells derived from *Azin2*-deficient mice showed reduced levels of spermidine and spermine, associated to decreased levels on intracellular and extracellular serotonin and increased histamine [120]. All these findings support a role for AZIN2 as regulator of biogenic amines such as serotonin and histamine in mast cells.

Later, in an extended analysis on the expression of AZIN2 in human tissues, the protein was also detected in several types of specific cells: Pulmonary type two pneumocytes, megakaryocytes, gastric parietal cells, acinar cells of sweat glands, in selected enteroendocrine cells, and different types of renal cells [121]. Remarkably, the transgenic *Azin2* knockout mice model provides an interesting tool to study the expression of AZIN2 protein in tissues or organs different to brain and testis in which moderated levels of *Azin2* mRNA had been detected. In fact, by using this strategy AZIN2 was shown to be also expressed in the endocrine pancreas and adrenal glands [95]. In these organs, AZIN2 was restricted to specific regions and cell types. In the adrenal gland, only the cells of the adrenal medulla displayed a positive X-Gal staining with a cytosolic and granular localization. Regarding to the pancreas, AZIN2 expression was located mainly in the islets of Langerhans, showing a heterogeneous pattern in the β-cells from unstained cells to cells showing strong granular and cytosolic staining. Interestingly, plasma insulin levels were significantly reduced in the *Azin2* knockout mice [95]. Altogether, these results support the idea that AZIN2 may have a role in secretory processes.

In spite of all these findings, the mechanism(s) by which AZIN2 might participate in the regulation of the functions of these specialized cells remains elusive. Its intracellular localization, associated to granular or vesicular structures, suggested that AZIN2 may act as a regulator of vesicular trafficking by locally activating polyamine biosynthesis [91]. Additionally, its proven effect antagonizing the effect of AZs on polyamine uptake would imply that AZIN2 could influence polyamine internalization and compartmentalization. In fact, elevated levels of polyamines are present in vesicular structures, like in mast cell secretory granules [122], although the mechanism for the vesicular uptake of polyamines is mostly unknown. A putative mechanism proposed the participation of a vesicular antiporter polyamine-proton for the vesicular sequestration of free cytosolic polyamines [123]. One can speculate that AZIN2 could have some direct or indirect effect on the regulation of both the plasma membrane polyamine transporter and the vesicular transporter, and hence the polyamine content of vesicular structures.

### 3.5. Gm853 as a New Paralogue of Odc/Azins with Leucine Decarboxylase Activity

Although AZIN1 and AZIN2 are two well-established ODC homologues with a widely expression in many different animal tissues, recent genomic studies predicted the existence of a new paralog of the family, that in mice received the name of *Gm853*. This murine gene is located in the same chromosome than *Azin2*, a characteristic shared by all their corresponding orthologs, suggesting that *Azin2* and *Gm853* presumably evolved from a common ancestor gene. However, overexpression of the gene in HEK293T revealed that the protein encoded by *Gm853* not only did not act as an antizyme inhibitor, but also presented enzymatic activity [124]. This is a not surprising fact since *Gm853* contains all the amino acid residues that are critical for the enzymatic activity of ODC [41]. Interestingly, the protein, which was mainly expressed in the mouse male kidney, was unable to decarboxylate ornithine or lysine, but instead was very active catalysing the decarboxylation of l-leucine to produce isopentylamine, an aliphatic monoamine with unknown biological function. The biological significance of this novel leucine decarboxylase remains to be elucidated.

## 4. Concluding Remarks, Controversies and Future Perspectives

As commented above, numerous in vitro experiments have clearly showed that both AZIN1 and AZIN2 share the capacity to interact with the three AZs, and as a result both proteins are able to increase polyamine biosynthesis and uptake. However, the physiological role of these two AZINs in mammalian cells appears to be quite different. Thus, whereas AZIN1 is required for normal embryonic development and clearly related with cell proliferation, AZIN2 presents a more restricted cellular distribution (differentiated cells) and is dispensable for embryonic development. Both abnormal AZIN1 expression and mRNA editing have been detected in different types of cancer, suggesting that AZIN1 may be considered as a potential carcinogenic molecule [81]. Besides, AZIN2, through the regulation of local intracellular levels of polyamines, might affect vesicle trafficking and secretory processes, although pathologies related to this protein have not been described so far.

**Figure 3:**
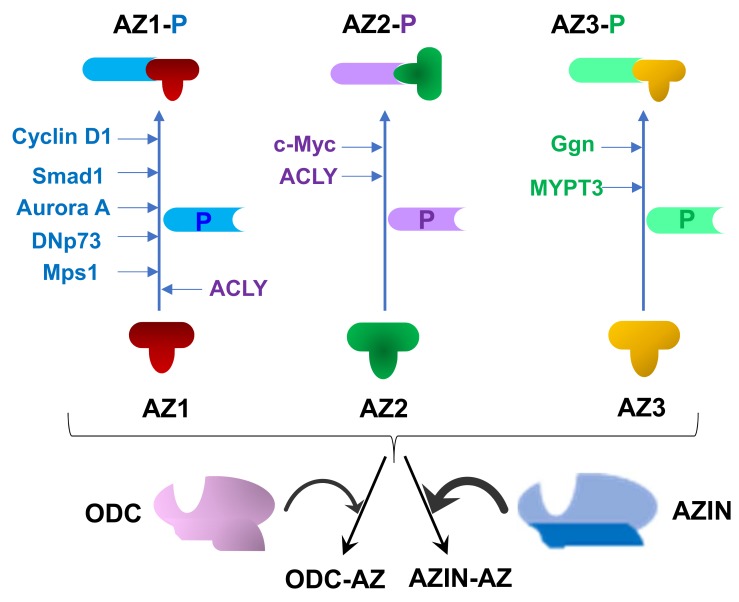
AZ-interacting proteins. The three AZs interact with ODC and AZINs, proteins implicated in polyamine metabolism. In addition, AZs also bind to other proteins not directly related with polyamines. In particular, AZ1 interacts with cell cycle proteins (cyclin D1, Smad, protein kinases such as Aurora A and Msp1), apoptosis related proteins (DNp73, an amino terminally truncated form of p73), or with the acetyl-CoA forming enzyme ATP citrate lyase (ACLY). AZ2 also interacts with ACLY and with the oncogenic protein c-Myc. AZ3 binds to the testicular protein gametogenetin (Ggn), and to MYPT3, a regulatory protein of protein phosphatases.

More recent evidence has revealed that AZs can bind to different molecular targets that do not belong to the polyamine metabolic pathway. Accordingly, AZINs, by negating the action of AZs on these targets, might exert polyamine independent effects. Several proteins, different to ODC or AZINs, have been identified as AZ-binding proteins (Figure 3). In some cases, the binding of the protein to AZ1 promoted its proteasomal degradation without ubiquitination. Several of these new targets of AZ1 are growth-related proteins such as Smad1, a transcriptional regulator of genes responsive to bone morphogenetic proteins [125,126]; cyclin D1, the activator of the cell cycle kinase CDK4 required for the transition G1/S [127]; the protein kinase Aurora A [128]; the mitotic check point kinase Mps1 [129], and the antiapoptotic DNp73, an amino terminally truncated form of the proapoptotic p73 [130]. All these new findings suggested that AZ1 might affect cell proliferation by polyamine-independent mechanisms. Other studies, however, did not find any significant effect of AZ1 or AZ2 on the degradation of cyclin D1, Aurora A or DNp73 in comparison with that of ODC by using a co-degradation assay [131]. In addition, they did not detect any antiproliferative effect of antizyme, when polyamine levels were maintained constant, suggesting that AZIN1 affects cell proliferation exclusively by affecting polyamine metabolism [131]. Nevertheless, some of the discrepancies might be related to the differential affinity of AZ1 towards their target proteins. In fact, a mechanistic study showed that cyclin D1 has a 4-fold lower binding affinity for AZ1 than does ODC and about 40-fold lower than AZIN1 [47]. It is then likely that in presence of ODC or AZIN1 the effect of AZ1 on the degradation of cyclin D1 would be negligible. In addition to AZ1, both AZ2 and AZ3 are also able to interact with specific proteins, affecting their degradation or modulating their activities. Thus, AZ2 binds to the oncogenic protein c-Myc and accelerates its proteasome-mediated degradation without ubiquitination [132]. AZ2 also interacts with ATP citrate lyase, the enzyme that catalyses the production of acetyl-CoA, a metabolite used for lipid biosynthesis or acetylation of cellular components [133]. In the case of AZ3, it has been reported that this testis specific AZ isoform can bind to other testicular proteins such as gametogenetin [114] and MYPT3, a regulator of protein phosphatases [115]. In all these cases, AZINs could indirectly modulate again the functions of these proteins. Finally, an emerging aspect on the possible additional regulatory activities of AZINs is related to the interaction of their mRNAs with certain micro RNAs (i.e., miR-433, miR-214), which may affect different biological processes such as fibrogenesis [66,84] or cardiac regeneration [119].

In conclusion, new efforts should be addressed to make progress in our understanding on the regulatory mechanisms that control AZINs expression as well as in the knowledge of the physiopathological repercussions of these proteins. In particular, although more studies are required for a better understanding of the implication of AZIN1 on cancer development, specifically the role of AZIN1 mRNA editing, targeting of AZIN1 for cancer therapy appears as a promising strategy that requires a rigorous validation.
